# The impact of positive, negative and neutral stimuli in a virtual reality cognitive-motor rehabilitation task: a pilot study with stroke patients

**DOI:** 10.1186/s12984-016-0175-0

**Published:** 2016-08-09

**Authors:** Mónica S. Cameirão, Ana Lúcia Faria, Teresa Paulino, Júlio Alves, Sergi Bermúdez i Badia

**Affiliations:** 1 Faculdade das Ciências Exatas e da Engenharia, Universidade da Madeira, Campus Universitário da Penteada, 9020-105 Funchal, Portugal; 2 Madeira Interactive Technologies Institute, Polo Científico e Tecnológico da Madeira, Caminho da Penteada, 9020-105 Funchal, Portugal; 3 Faculdade de Psicologia e de Ciências da Educação da Universidade de Coimbra, Coimbra, Portugal

**Keywords:** Emotional stimuli, Stroke, Virtual Reality, Cognitive and motor rehabilitation, Valence, Eye tracking

## Abstract

**Background:**

Virtual Reality (VR) based methods for stroke rehabilitation have mainly focused on motor rehabilitation, but there is increasing interest in integrating motor and cognitive training to increase similarity to real-world settings. Unfortunately, more research is needed for the definition of which type of content should be used in the design of these tools. One possibility is the use of emotional stimuli, which are known to enhance attentional processes. According to the Socioemotional Selectivity Theory, as people age, the emotional salience arises for positive and neutral, but not for negative stimuli.

**Methods:**

For this study we developed a cognitive-motor VR task involving attention and short-term memory, and we investigated the impact of using emotional images of varying valence. The task consisted of finding a target image, shown for only two seconds, among fourteen neutral distractors, and selecting it through arm movements. After performing the VR task, a recall task took place and the patients had to identify the target images among a valence-matched number of distractors. Ten stroke patients participated in a within-subjects experiment with three conditions based on the valence of the images: positive, negative and neutral. Eye movements were recorded during VR task performance with an eye tracking system.

**Results:**

Our results show decreased attention for negative stimuli in the VR task performance when compared to neutral stimuli. The recall task shows significantly more wrongly identified images (false memories) for negative stimuli than for neutral. Regression and correlation analyses with the Montreal Cognitive Assessment and the Geriatric Depression Scale revealed differential effects of cognitive function and depressive symptomatology in the encoding and recall of positive, negative and neutral images. Further, eye movement data shows reduced search patterns for wrongly selected stimuli containing emotional content.

**Conclusions:**

The results of this study suggest that it is feasible to use emotional content in a VR based cognitive-motor task for attention and memory training after stroke. Stroke survivors showed less attention towards negative information, exhibiting reduced visual search patterns and more false memories. We have also shown that the use of emotional stimuli in a VR task can provide additional information regarding patient’s mood and cognitive status.

## Background

According to the World Health Organization, fifteen million people worldwide suffer a stroke each year, leaving 5 million survivors permanently disabled. For those who survive, reducing the impact of post stroke impairment is a major goal [1]. It has been estimated that more than 70 % of individuals experience some degree of cognitive decline in the first few weeks following stroke and that more than one third remain cognitively impaired even 1 year post stroke [2]. These cognitive impairments have a direct influence on patients’ quality of life, being associated with greater rates of institutionalization [3] and higher health-care costs [4].

Among the most frequent sequels, post stroke patients commonly present decreased executive functioning, mental slowing, and impairment of goal formulation, initiation, planning, organizing, sequencing, executing, abstracting, and attention [1], being also at risk of developing dementia [2, 5]. Although several screening tools are available and are generally administered, specific deficits are only detectable with more complete neuropsychological assessments, which are rarely performed [6]. Moreover, most assessments are paper-and-pencil based and are not performed in the context of meaningful real world tasks, thus they may miss important information. Consequently, there is a need to develop and employ more insightful assessment tools. This would allow the prescription of intensive cognitive and motor rehabilitation programs precisely tailored to the needs of patients, hence maximizing gains and transference of those to real-world tasks.

### Virtual Reality (VR) as an assessment and rehabilitation tool

Recent research has shown that VR can be used in the assessment of motor and cognitive function by using simulations that relate to real-world skills [7–10]. Current assessment methods lack this aspect and evaluation in real-world context is costly and many times impracticable. Instead, VR has been applied to assess motor function in the context of ADLs [11] as well as cognitive functions in the context of a virtual city [7]. In fact, VR neuropsychological assessment tools have been also validated against traditional methods [8, 12], holding promise for the future of neuropsychological assessment. Moreover, enriched virtual environments have the potential to optimize rehabilitation by manipulating practice conditions that explicitly engage motivational, cognitive, motor control and sensory feedback-based learning mechanisms [13]. Unfortunately, most VR rehabilitation approaches are generally dedicated either to motor or cognitive rehabilitation aspects. Nevertheless, given the dual motor and cognitive components of ADL, a combined motor and cognitive VR approach could provide training more consistent with real-world settings [14]. In fact, a recent meta-analysis identified a moderate association (*r* = 0.43) suggestive of interdependency between cognitive and motor recovery in stroke survivors [15]. This has also been observed in a study with stroke survivors [16, 17] that used a VR adaptation of the widely used Toulouse Piéron (TP) cancellation task [18]. Using VR adaptations of standardized assessment instruments is particularly interesting because it allows direct comparison with the paper-and-pencil counterpart.

### Eye gaze during action execution in VR

There is strong evidence showing that motor areas are being engaged not only during motor execution but also during the observation of motor actions [19–21]. Eye gaze is closely linked to prediction and motor control, and has been used to study the neural mechanisms underlying the observation and execution of actions [22, 23]. Based on the premise of shared neural mechanisms for execution and observation, researchers have shown that action observation can have a positive impact in the rehabilitation of motor function after stroke [24, 25]. Recent studies on gaze metrics of healthy participants during action execution and observation have provided further evidence that, both execution and observation, partially share underlying neural processes [26]. A study combining VR and eye tracking with stroke survivors and healthy participants has shown that movement metrics in the observation of a VR reaching task are sensitive to motor impairment [23]. A previous study using the same system also identified differences in stroke survivors between action execution and observation, and between paretic and non-paretic arms during observation of motor actions but not during action execution [27]. Given the emergence of novel low cost eye tracking devices, the combination of eye tracking technology and VR has large potential to be used in stroke rehabilitation to inform about underlying mechanisms during VR training.

### The role of emotional stimuli in rehabilitation

Despite the wealth of evidence concerning the value of VR for rehabilitation of stroke patients [28], there is surprisingly little research about the type of content being used (neutral, abstract, emotional, etc.). One particularly interesting case is the use of emotional stimuli with different valences. Affective valence refers to the pleasantness of a given stimulus, with positive and negative valence indicating attractive and aversive stimuli, respectively. Stimuli of neutral valence are commonly perceived as having no or weak valence (positive or negative) [29].

From the literature with healthy participants we know that emotional stimuli are remembered better and more vividly than non-emotional stimuli [29]. This phenomenon, known as the emotional enhancement of memory, has been replicated across a range of paradigms and stimulus types. These emotional enhancements in memory are, at least, partly due to the increased attention directed toward emotional items at encoding [30]. However, emotional items are often remembered at the expense of their contexts, this is, peripheral features of visual scenes are remembered less when an emotional item is present in the scene than when only non-emotional items are present. The rationale behind this phenomenon is that items with a high affective valence tend to capture attention and to get prioritized in the processing chain [31]. Since more attentional resources are directed towards the emotional components, people seem more likely to encode the emotional components of the scene and less likely to encode neutral contextual information.

The processing of emotional stimuli seems also to be affected by age [32]. In fact, the Socioemotional Selectivity Theory states that there is an age-associated motivational shift towards emotional goals [33]. This theory states that when emotional material is attended to, is weighed more heavily, processed more deeply, and better remembered than non-emotional material. Recent evidence shows that the recall of emotional information is disproportionately positive as people age [34]. These results are consistent with eye tracking research that showed that when a negative and a neutral picture are displayed together, both young and older adults initially glance at the negative picture but young adults look for a longer time at the negative picture [35].

It has also been found that emotional stimuli can induce more false memories than non-emotional stimuli in healthy individuals, with stimuli of negative valence being more often falsely remembered when compared to stimuli of positive or neutral valence [36]. The apparent paradox, that negative emotions can simultaneously improve and impair memory by its high valence, has been consistently found in memory recall experiments, inclusively in participants with major depression [37]. One hypothesis is that negative valence causes a narrowing of attention such that, spatial and temporal information associated with the emotional item, are better attended to and later remembered, while peripheral information is likely to be forgotten. An example is the *weapon focus effect*, where there is enhanced memory for a weapon in a scene but reduced memory for details of the background [38]. This focus may lead to selective memory for emotional components [39]. However, some researchers argue that selective memory of emotional content is not strongly related to attention at encoding [40].

In this paper we present a pilot study with a VR based cognitive-motor task that consists of searching and selecting target images within a pool of distractors. Target images can be pleasant (positive valence), unpleasant (negative valence) and neutral. The primary objective of this work is to investigate the impact of emotional content in the performance of cognitive-motor rehabilitation training through performance and eye tracking data. Specifically, to determine how valence of stimuli affects task performance (attention) and recall (memory). We have the following hypotheses:

1. The valence of the pictures will have an effect in VR task performance, with lower performance for negative pictures and higher performance for positive pictures when compared to neutral targets.
2. The valence of the pictures will have an effect in recall, with negative pictures generating more false memories when compared to positive and neutral images.

As secondary objectives, we 1) study how the cognitive profile of stroke patients modulates performance in tasks using emotional content; and 2) assess the feasibility of the proposed VR rehabilitation paradigm that integrates both cognitive and motor domains for improved transference to real-world settings.

## Methods

### Participants

Participants were recruited at the Nélio Mendonça and João Almada Hospitals (Madeira Health Service, Portugal), based on the following inclusion criteria: ischemic stroke; normal or corrected-to-normal vision; capacity to be seated; non-aphasic and with sufficient cognitive ability to understand the task instructions, as assessed by the clinicians. The sample consisted of ten (7 female, 3 male) middle-aged (54.2 ± 9.2 years old) stroke survivors (1 right hemisphere, 8 left hemisphere and 1 cerebellum), at 16.6 ± 19.5 months post stroke, and 8.1 ± 5.8 years of schooling. 6 had some computer literacy. The study was approved by the Madeira Health Service - SESARAM Ethical Committee (approval number 47/2013) and all the participants gave their informed consent.

### Setup

**Fig. 1:**
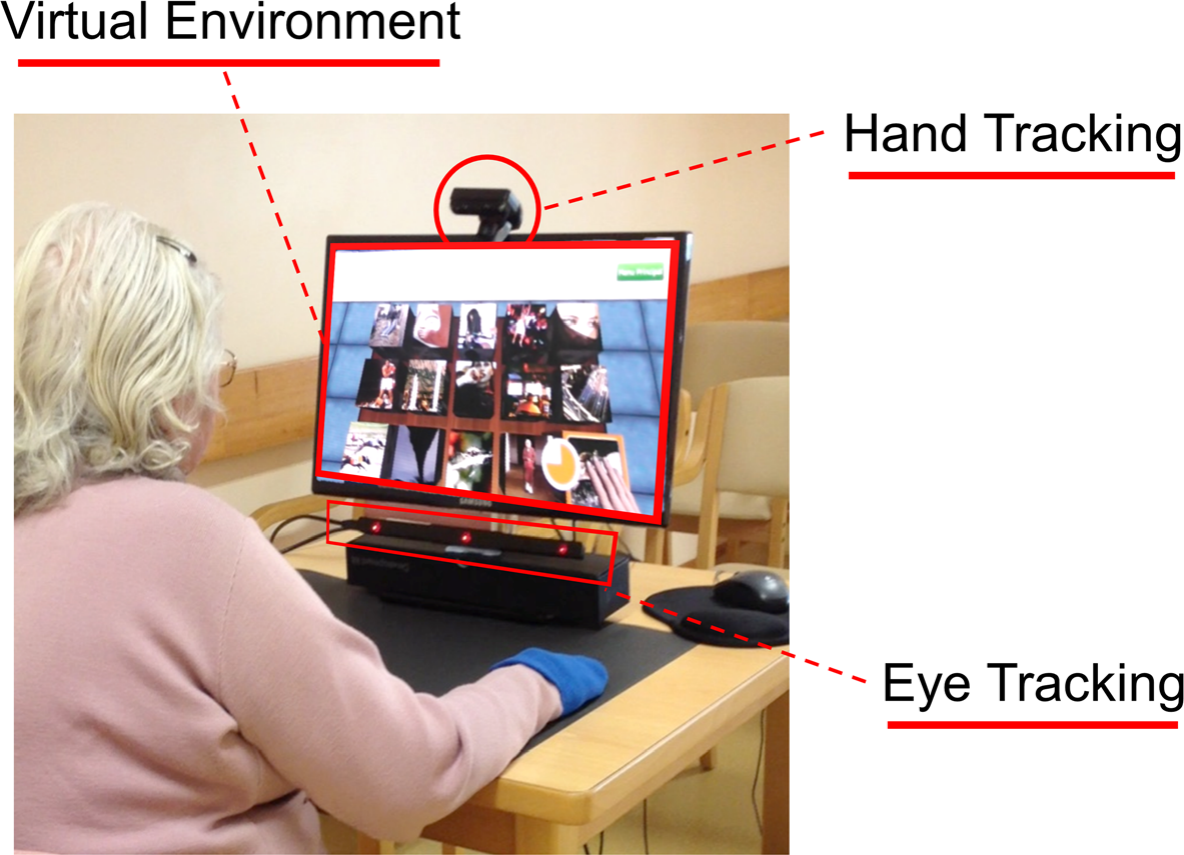
Experimental setup. The setup includes an eye-tracker, hand tracker and a virtual environment displayed on a high-resolution display monitor. Patients interact with the system through arm movements on a flat surface

The setup consists on a 37.7 cm × 26.5 cm high-resolution display monitor (1920 × 1068 pixels), a Sony PlayStation Eye (Sony Interactive Entertainment, San Mateo, California, USA) for tracking arm movements and an eye-tracker (Tobii® EyeX, Tobii Technology, Stockholm, Sweden) for recording eye movements (Fig. 1). The eye-tracker works at distances from 45 to 100 cm, samples data at 30–60Hz and has a latency of 15 ms +/− 5 ms. The custom VR task was developed using the Unity 3D game engine (Unity Technologies, San Francisco, USA). The system was implemented on a PC running Windows 7.

#### Motor component

Participants were seated at a distance of ~55 cm from a 21.5 inch monitor with a total height of ~41 cm positioned on the table, and rested both arms on a tabletop. The task consisted on performing two-dimensional movements on the surface of the table with a single arm. These enabled participants with no force against gravity to perform the task. Most participants used their paretic arm to perform the task; participants without sufficient motor capacity used their unaffected arm (3/10 participants). The arm used for the interaction wore a colored glove that allowed real arm movements to be captured through a camera-based color tracking software (AnTS) [41] and mapped onto the movements of a virtual arm. The VR environment had a built-in calibration function to compute the active range of motion as described in [42]. This calibration matches the maximum physical range of motion of the participant to the maximum range of motion required in the VR task, normalizing the required motor effort to the skill set of the user. The participant controls a virtual representation of the arm to complete the task.

#### Cognitive component

The VR task was designed as a VR adaptation of the TP task (TP-VR) [16]. This task was extended to also incorporate emotional stimuli. A target image of 21 × 21 cm^2^ is presented in the center of the screen. Immediately after, in a 3D environment, fifteen cubes with images are displayed in a 3x5 grid structure on top of a table. From these fifteen images, one is the target and fourteen are distractors, randomly selected from a set of 92 neutral valence images or from the set of 8 TP symbols.

### Emotional stimuli pictures

The pictures were selected from the International Affective Picture System (IAPS) [43]. This widely used picture set, that has also been used in studies with stroke survivors [44–46] , consists of photographs of people, animals, objects, and scenes that have been originally rated through the 9-points Self-Assessment Manikin (SAM) along the dimensions of affective valence (ranging from unpleasant to pleasant) and arousal (ranging from calm to excited). 182 images were selected for the purpose of this study. The categorization of the images as positive, negative and neutral was based on the original valence and arousal scores provided by the IAPS. Our selection of neutral, negative and positive images had valence scores in the range of 4.5–5.5, 1.66–2.58 and 7.53–8.34, respectively. A Friedman test confirmed that valences were significantly different across conditions (χ^2^ (2) = 28.0, *p* < 0.001). Arousal of the images was kept neutral with scores between 4.5 and 5.5.

### Study design and protocol

A within-subjects design was used with three experimental conditions corresponding to 3 types of stimuli (positive, negative, and neutral). In addition, the abstract stimuli of the original TP task were also used for comparison with the paper-and-pencil TP task, since this is a well-established attention assessment tool.

Before starting the experiment, participants went through an average of five training trials only with abstract stimuli (TP-VR). The training was intended to provide a clear understanding of the VR task and valence rating, as well as to get used to the natural user interface (AnTS) [17].

**Fig. 2:**
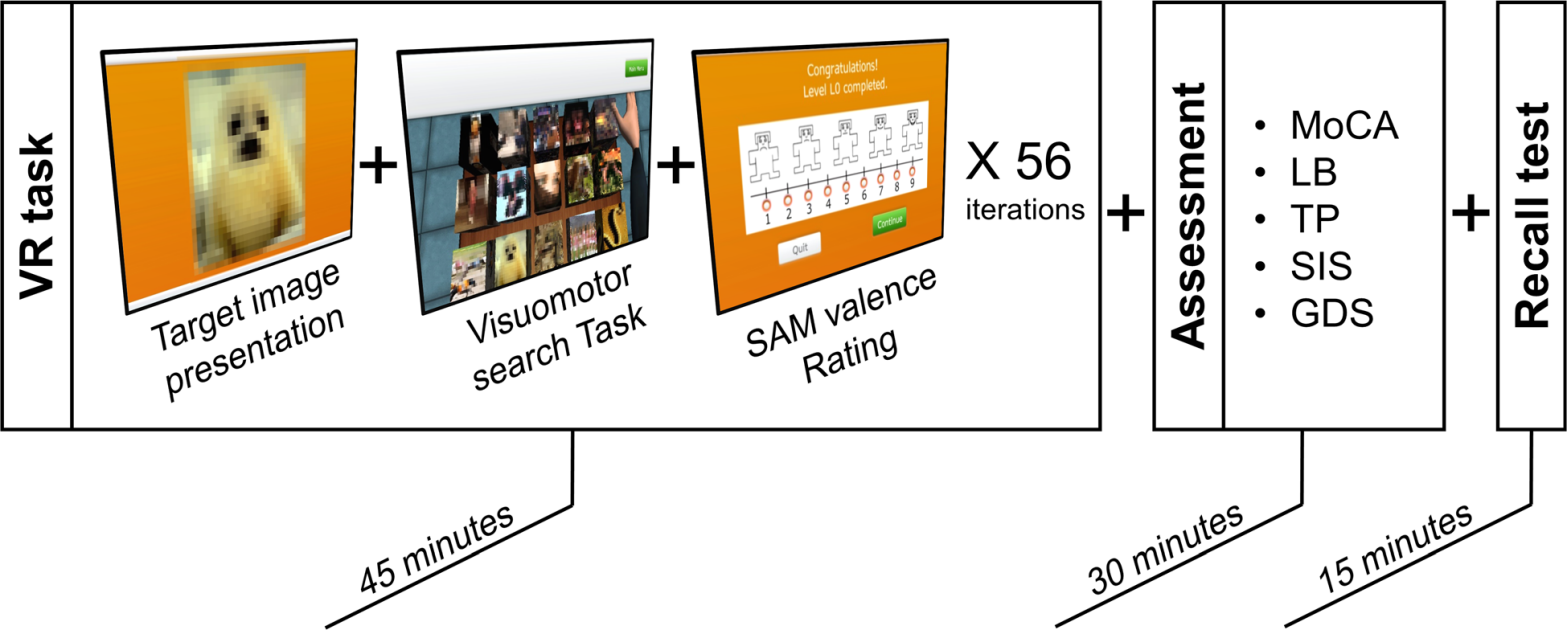
Experimental protocol. The protocol consisted of the performance of a cognitive-motor VR task, followed by an assessment and a recall test

The experiment was single-session and entailed 56 trials, including the 3 different conditions plus TP trials presented in an alternating order (Fig. 2). For each trial, a target image was presented for 2 s and then had to be selected among a pool of distractors. Each trial ended with a computerized version of the 9-point SAM, through which the user indicated how he/she felt about the target image, where 1 is very sad and 9 is very happy. There was no time limit to perform each trial, but in average the 56 trials were performed in approximately 45 min. A recall test took place 30 min after the end of the VR task. It included 90 images from which 30 had positive valence (14 targets + 16 distractors), 30 had negative valence (14 targets + 16 distractors) and 30 had neutral valence (14 targets + 16 distractors). Images were randomized and distributed within ten A3 pages (9 images per page). Participants indicated the ones they thought they had previously seen in the VR task. The cognitive assessment of the users was performed during the 30 min between the end of the VR task and start of the recall task (Fig. 2). The training was run by a researcher and the assessment and recall task by a neuropsychologist.

### Assessment

The assessment was done by a trained neuropsychologist through a number of standard assessment instruments to have a comprehensive cognitive profile of the users (Table 1). The cognitive screening was made through the Montreal Cognitive Assessment (MoCA) [47], which addresses the following domains: Executive Functions, Naming, Attention, Language, Reasoning, Memory and Orientation. The Line Bisection (LB) test [48] was used to assess possible neglect. A short version of the TP test [49] was also included to compare the performance on paper-and-pencil and the performance on the virtual environment. To assess the subjective impact of stroke, we used the following selected dimensions of the Stroke Impact Scale v3.0 [50]: Strength, Hand function, Memory, Communication and Recovery. Acknowledging the effects that different states of mood (e.g. depression) can have on cognition, we assessed depressive symptomology through the Geriatric Depression Scale – 30 (GDS-30) [51]. The tests were delivered in the following order: MoCA, LB, TP, SIS 3.0 and GDS-30.

**Table 1:** Demographics and clinical profile of the participants

ID	Sex (M/F)	Age	Months post stroke	Stroke side (L/R/C)	MoCA (Max = 30)	LB (I/N)	TP (Max = 100)	SIS strength (Max = 100)	SIS hand (Max = 100)	SIS communication (Max = 100)	SIS memory (Max = 100)	SIS recovery (Max = 100)	GDS-30 (Max = 30)
P1	F	55	2	R	22	N	100	56.3	40	100.0	92.9	50	3
P2	F	41	36	L	24	N	90	50.0	0	82.1	82.1	65	9
P3	F	74	5	L	15	N	60	62.5	60	82.1	67.9	70	10
P4	F	47	3	L	9	N	10	68.8	40	92.9	75.0	70	15
P5	M	55	11	L	13	N	60	68.8	70	92.9	71.4	70	5
P6	F	50	42	L	8	N	30	56.3	5	21.4	100.0	80	4
P7	M	61	53	L	16	N	20	31.3	0	60.7	78.6	70	3
P8	F	47	1	C	25	N	100	100.0	100	96.4	89.3	90	5
P9	M	59	1	L	25	N	80	18.8	75	100.0	85.7	6	11
P10	F	53	12	L	17	N	30	50.0	20	57.1	60.7	40	23
N = 10	3/7	54.2 ± 9.2	16.6 ± 19.5	8/1/1	17.4 ± 6.4	0/10	58.0 ± 33.9	56.3 ± 22.0	41.0 ± 34.9	78.6 ± 25.2	80.4 ± 12.1	61.1 ± 23.9	8.8 ± 6.4

The table shows sex with *M* male and *F* female; stroke side with *L* left, *R* right and *C* cerebellum; and the results on the LB test with *I* impaired and *N* normal. The descriptive statistics show the mean and the standard deviation

### Data analysis

From the VR task performance data, we extracted for each condition the percentage of correct target selection, resulting from the performance of both motor and cognitive components of the task; the mean task completion time per participant; and valence of the images as rated by the participants. From the recall task, we computed the recall performance as the percentage of correctly recalled images (true positives) per valence condition (neutral, positive, negative); the percentage of wrongly recalled images (false positives); and the total number of errors in the recall task (missed images/false negatives plus wrongly identified images/false positives). We tested for differences across conditions for the different dependent variables. To understand how performance in the VR and recall tasks could be explained as a function of the profile of the participants, we ran linear regressions for the results obtained under each condition, using the MoCA and GDS scores as factors in our models. In addition, the Pearson and Spearman coefficients were computed to search for meaningful correlations between variables for parametric and nonparametric data respectively.

Eye tracking data was temporally smoothed with a squared Savitzky-Golay FIR smoothing filter to data frames of length 29, and converted from screen coordinates (X, Y) to degrees. Resting periods and segments with missing or corrupted data were removed from the analysis. According to the velocity (*v*) profile of the data, eye movements were classified into 1) fixations (*v* < 5 deg/s); 2) saccadic movements (*v* > 30 deg/s), and 3) smooth pursuit (5 < *v* < 30 deg/s) consistent with [52, 53]. For each behavior detected, the number of occurrences and their duration were assessed. In addition, the total eye gaze trajectory length (in pixels) and its dispersion (standard deviation) were extracted. Heat maps to determine the density of the occurrence of the different eye gaze events were generated on a 10 × 10 grid of approximately 4.3° resolution centered at the target location, and smoothed using cubic interpolation.

For each variable, the normality of the distribution was assessed using the one sample Kolmogrov-Smirnov test. Because most distributions deviated from normality, non-parametric statistical tests were used for the analysis. For assessing the overall difference between experimental conditions, a Friedman test was used. For pairwise comparisons, the Wilcoxon’s T matched pairs signed ranks test was used for related measures, with a Bonferroni correction to account for the number of comparisons. One-tailed tests were used when hypotheses were directional. Data were analyzed using Matlab (MathWorks Inc., Natick, MA, USA) and the Statistical Package for the Social Sciences 20 (SPSS.20).

## Results

### Primary objective - Effect of emotional content on VR task performance and eye gaze behavior

#### Does emotional content affect VR task performance?

**Fig. 3:**
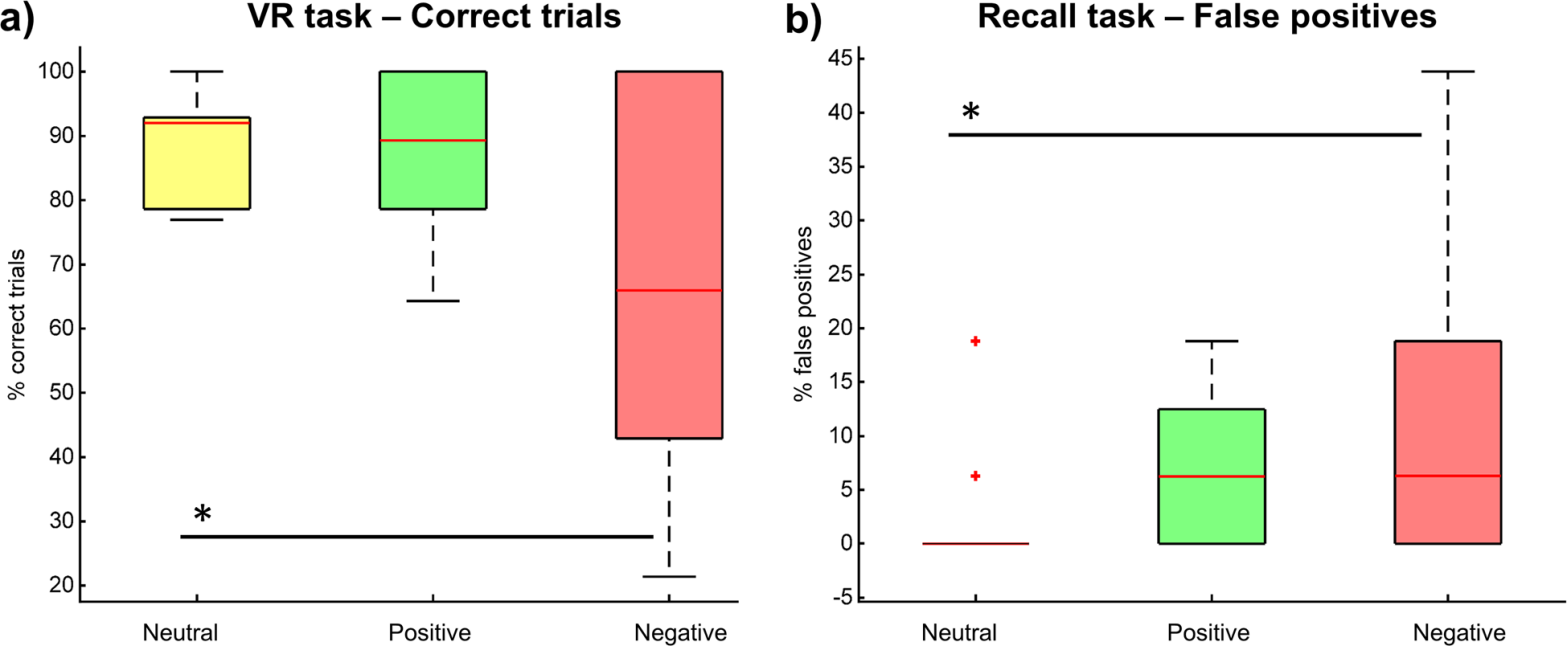
Performance in VR and recall tasks. Performance versus emotional content of visual stimuli in terms of (**a**) percentage of correct answers in the VR task, and (**b**) percentage of false positive memories in the memory recall task. * indicates *p* < 0.05/2

In order to assess the impact of using emotional content in the VR task, we compared the performance in the 3 valence conditions in terms of the mean percentage of correct responses and completion times. In terms of the percentage of correct target selection, we found a significant effect across conditions (Fr(2) = 6.00, *p* = 0.05) (Fig. 3a). We hypothesized that the percentage of correct target selection would be lower for negative targets and higher for positive targets, when compared to neutral targets. Planned comparisons of the performance for neutral targets (Mdn = 92.1 %, IQR = 16.1) with that for positive (Mdn = 89.3 %, IQR = 22.3) and negative (Mdn = 65.95 %, IQR = 60.1) targets showed that the performance for negative targets was significantly lower (T = 1.0, *p* < 0.05/2, one-tailed); but there was no difference in the percentage of correct selections between positive and neutral targets (T = 3.0, *p* > 0.05/2, one tailed). Concerning the time to completion, no significant differences were found across positive (Mdn = 14.43 s, IQR = 8.41), negative (Mdn = 14.54 s, IQR = 8.52) and neutral (Mdn = 15.33 s, IQR = 10.69) stimuli conditions (Fr(2) = 2.60, *p* > 0.05).

#### Does emotional content impact eye gaze?

**Fig. 4:**
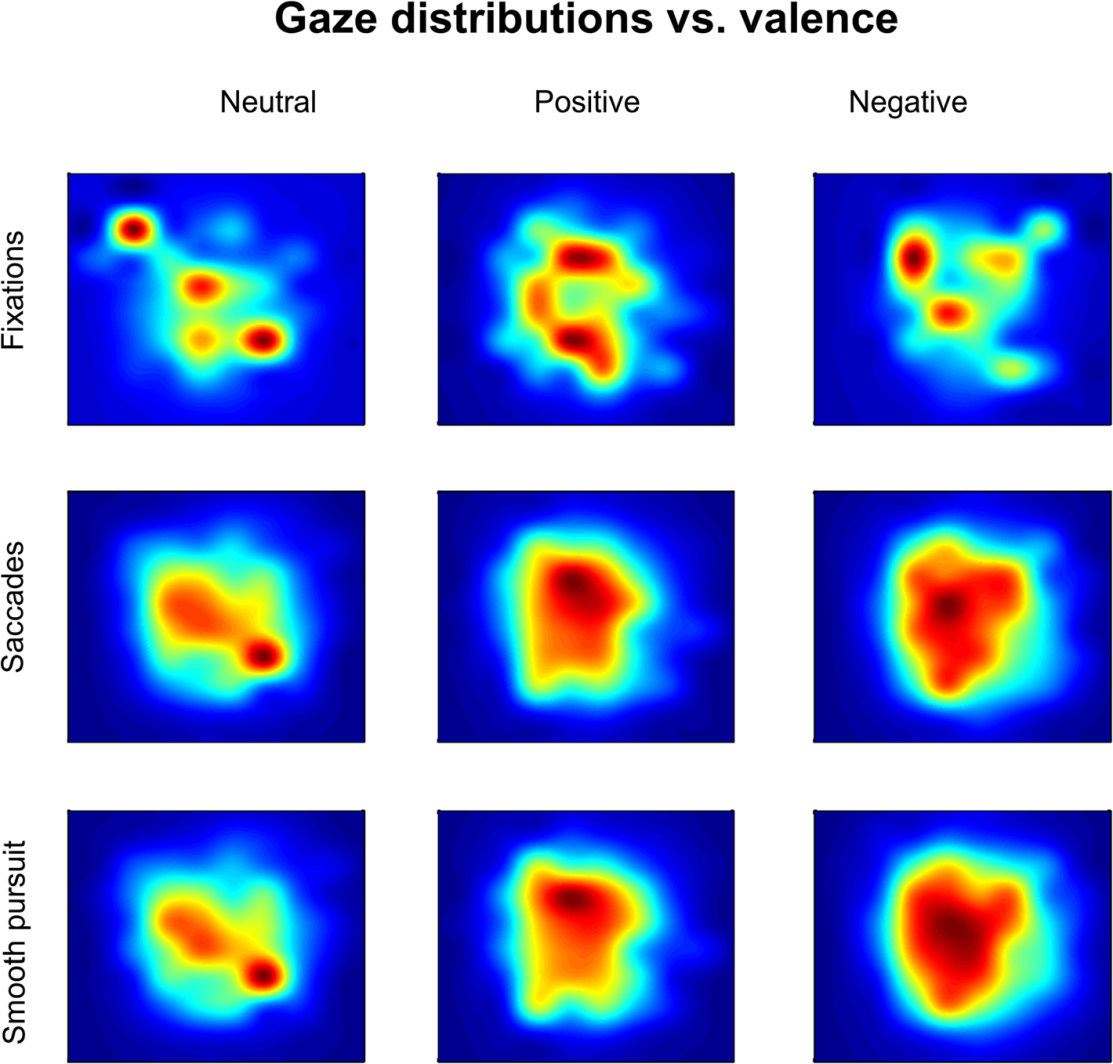
Eye gaze for all trials (correct and incorrect). Event density maps for fixations, saccades and smooth pursuit depending on the emotional valence of the stimulus. Map center position indicates the position of the target stimulus on the computer screen

Event density maps for fixations, saccades and smooth pursuit, when all trials (correct and incorrect) are considered, revealed dissimilar gaze patterns depending on the valence of the presented stimulus (Fig. 4). In these maps the central point is the position of the target image. A repeated measure analysis using the interquartile range of the data (dispersion of the distribution) in X and Y as dependent variables showed a significant effect across conditions for fixations (X: Fr(2) = 7.8, *p* < 0.05; Y: Fr(2) = 15.8, *p* < 0.001), saccades (X: Fr(2) = 15.8, *p* < 0.001; Y: Fr(2) = 13.4, *p* < 0.01) and smooth pursuit (X: Fr(2) = 15.8, *p* < 0.001; Y: Fr(2) = 13.4, *p* < 0.01). We can observe that eye gaze is less centered on the target image for neutral stimuli than for positive and negative stimuli. Particularly, saccades and smooth pursuit are more aligned with the target position for positive and negative valence and more peripheral for neutral content.

**Fig. 5:**
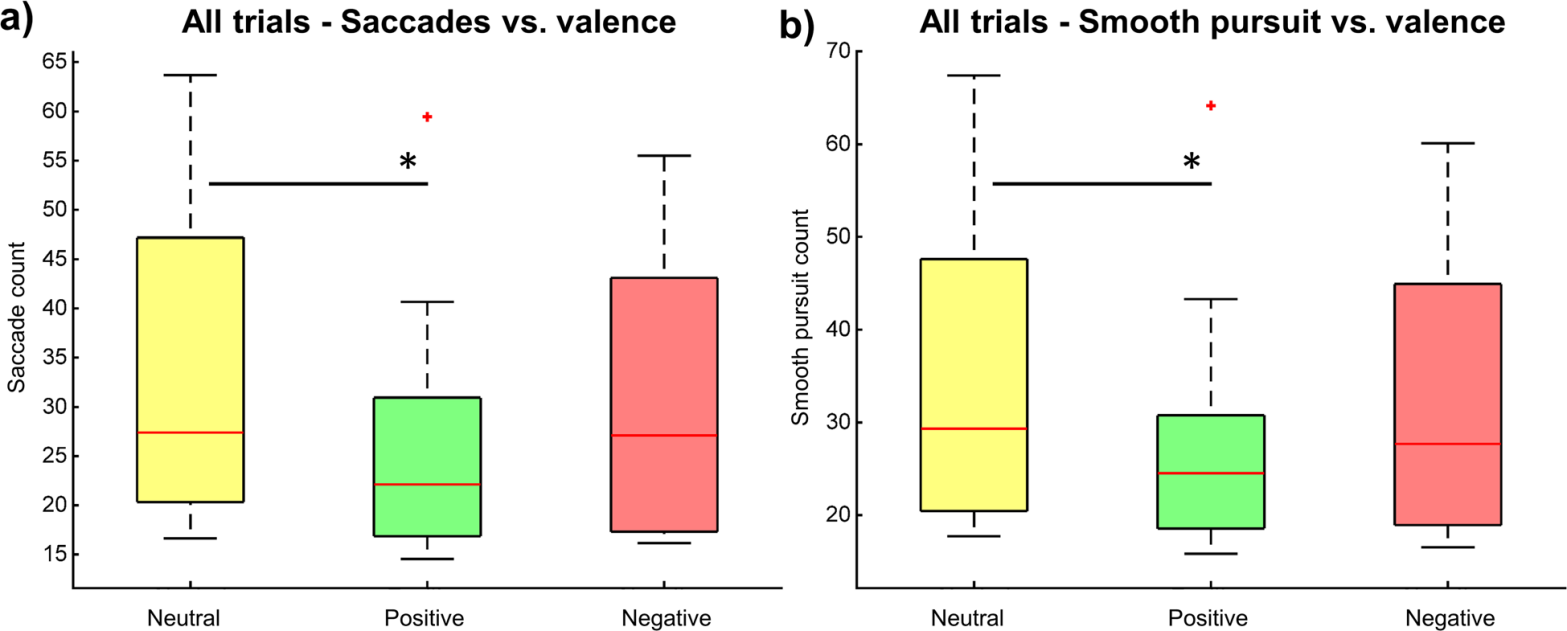
Effect of valence on saccades and smooth pursuit. **a** saccades count. **b** smooth pursuit count. * indicates *p* < 0.05/3

More specifically, the quantification of the effect of valence of the target image on the count and duration of eye tracking metrics for all trials (Table 2, All trials) revealed a significant effect across conditions for saccade count (Fr(2) = 9.38, *p* < 0.01) and smooth pursuit count (Fr(2) = 8.6, *p* < 0.05). Specifically, pairwise comparisons showed that the saccade count for positive images (Mdn = 22.1, IQR = 14.1) was lower than for neutral (Mdn = 27.4, IQR = 26.8) and negative images (Mdn = 27.1, IQR = 25.8). However, only the positive-neutral comparison was significant (T = 1.0, *p* < 0.05/3) (Fig. 5a). For smooth pursuit count, again the number of counts was less for positive (Mdn = 24.5, IQR = 12.3) than for neutral (Mdn = 29.3, IQR = 27.2) and negative (Mdn = 27.7, IQR = 26.0) images, and only the positive-neutral comparison was significant (T = 3.0, *p* < 0.05/3) (Fig. 5b). The median eye trajectory length (in pixels) and trajectory standard deviation were not significantly different across conditions.

**Table 2:** Median and (IQR) of eye tracking metrics for neutral, positive and negative valence

	All trials				Correct trials				Incorrect trials			
	Neutral	Positive	Negative	p	Neutral	Positive	Negative	p	Neutral	Positive	Negative	p
Fixation count	3.1 (1.0)	3.3 (1.6)	2.8 (1.4)	0.8357	1.9 (0.8)	1.8 (0.6)	1.5 (1.0)	0.3320	1.2 (1.0)	2.0 (1.6)	1.1 (1.0)	0.0705
Fixation duration (ms)	70.0 (45.0)	94.3 (48.2)	83.0 (36.2)	0.7408	50.4 (18.3)	47.2 (17.1)	56.3 (22.7)	0.9048	33.4 (13.2)	40.4 (23.3)	57.9 (21.8)	0.0536
Saccade count	27.4 (26.8)	22.1 (14.1)	27.1 (25.8)	**0.0092**	26.9 (23.7)	22.1 (11.6)	23.9 (13.2)	0.1319	46.3 (33.8)	40.4 (25.5)	42.6 (13.6)	**0.0183**
Saccade duration (ms)	357.6 (35.3)	388.1 (107.7)	352.7 (59.5)	0.1225	358.6 (45.3)	394.1 (94.3)	352.9 (60.6)	0.1225	345.2 (23.4)	353.4 (44.0)	323.8 (46.4)	1.0000
Smooth pursuit count	29.3 (27.2)	24.5 (12.3)	27.7 (26.0)	**0.0136**	29.0 (25.1)	23.7 (10.2)	25.1 (14.8)	0.1225	47.3 (32.7)	38.8 (24.4)	43.5 (16.1)	0.0622
Smooth pursuit duration (ms)	274.4 (33.1)	303.2 (59.1)	269.1 (43.4)	0.2725	274.3 (36.8)	308.8 (57.7)	275.4 (53.6)	0.3012	267.5 (29.6)	263.4 (107.6)	251.6 (35.7)	0.6514
Trajectory length (px)	273.3 (49.6)	263.4 (58.1)	265.8 (41.1)	0.6703	255.5 (63.8)	263.2 (56.7)	259.3 (25.3)	0.6703	372.8 (63.8)	237.4 (183.3)	303.3 (70.8)	**0.0183**
Trajectory Std (px)	162.4 (30.2)	148.9 (29.0)	149.7 (29.5)	0.4066	152.6 (28.4)	152.8 (26.8)	149.5 (51.2)	0.9048	204.6 (23.8)	185.1 (79.6)	142.6 (58.6)	**0.0498**

The *p* values are the probability values in the Friedman test. Bold indicates significant differences

#### Is eye gaze modulated by the correctness of choices?

**Fig. 6:**
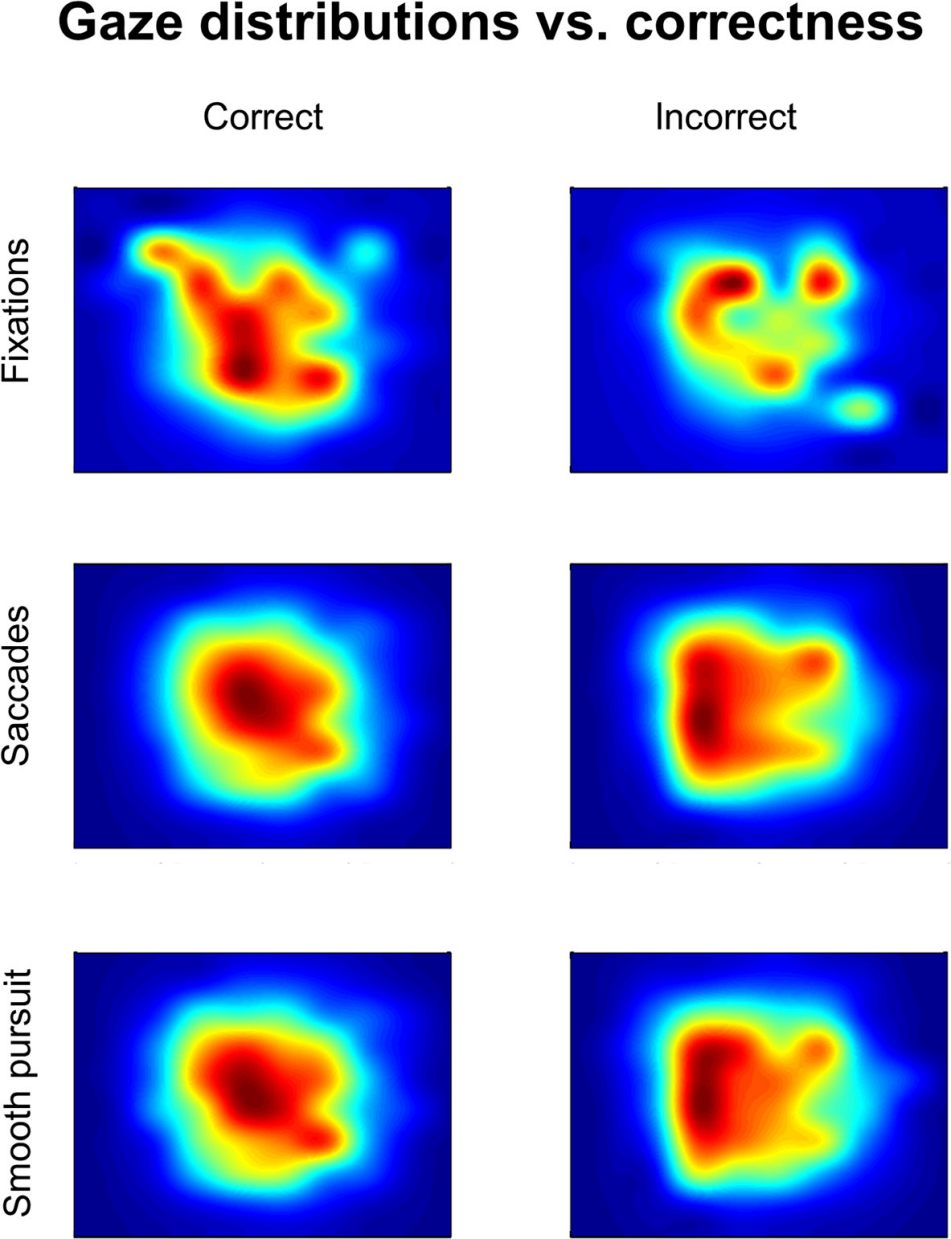
Eye gaze for correct and incorrect trials. Event density maps for fixations, saccades and smooth pursuit depending on the correctness of the choice. Map center position indicates the position of the target stimulus on the computer screen

**Fig. 7:**
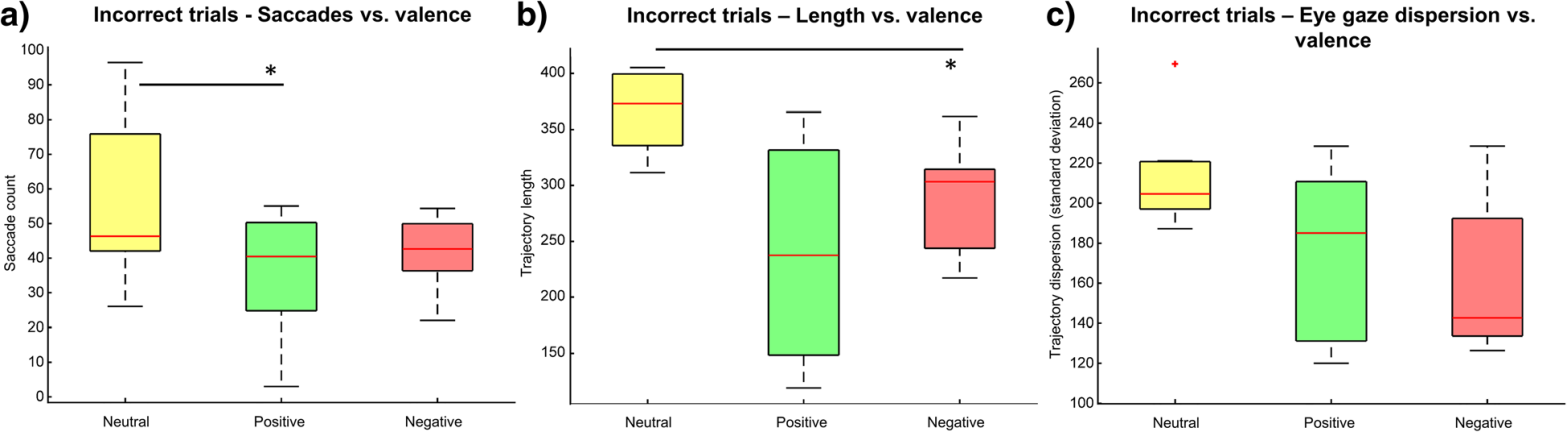
Effect of valence on eye tracking metrics for incorrect trials. **a** saccades count. **b** trajectory length. **c** dispersion. * indicates *p* < 0.05/3

When analyzing eye gaze for correct and incorrect trials separately, we observe that the patterns are different. As expected, eye gaze is more centered around the target image for correct trials and more peripheral for incorrect trials (Fig. 6). When addressing only the correct trials we found no significant differences in any of the eye tracking metrics across the neutral, positive and negative conditions (Table 2). Differences across conditions arise from incorrect trials. There were significant differences across conditions for saccade count (Fr(2) = 8.0, *p* < 0.05). Consistent with the previous results, pairwise comparisons showed that the number of saccades for positive images (Mdn = 40.4, IQR = 25.5) was lower than for neutral (Mdn = 46.3, IQR = 33.8) and negative (Mdn = 42.6, IQR = 13.6) images. Again, only the positive-neutral comparison was significant (T = 0.0, *p* < 0.05/3) (Fig. 7a). Trends were found for fixation count (Fr(2) = 5.3, *p* = 0.0705), fixation duration (Fr(2) = 5.85, *p* = 0.0536) and smooth pursuit count (Fr(2) = 5.56, *p* = 0.0622).

Concerning the length of eye gaze trajectories, we identified differences for trajectory length (Fr(2) = 8.0, *p* < 0.05) and trajectory dispersion (standard deviation) (Fr(2) = 6.0, *p* < 0.05). Trajectories were shorter for positive images (Mdn = 237.4px, IQR = 183.3) when compared to neutral (Mdn = 372.8px, IQR = 63.8) and negative images (Mdn = 303.3px, IQR = 70.8). However, only the negative-neutral medians were significantly different (T = 0.0, *p* < 0.05/3) (Fig. 7b). Finally, for the dispersion of the trajectory as measured by its standard deviation, although there was less variability for negative images (Mdn = 142.6px, IQR = 58.6) than for positive (Mdn = 185.1px, IQR = 79.6) and neutral images (Mdn = 204.6px, IQR = 23.8), these differences were not considered significant after Bonferroni correction (*p* > 0.05/3) (Fig. 7c).

### Primary objective - Effect of emotional content on memory recall

Concerning the recall task, we did not find significant differences between the overall recall performance across conditions (Fr(2) = 0.0, *p* > 0.05). Interestingly, when considering only the wrongly identified images (false memories) we found significant differences across conditions (Fr(2) = 7.58, *p* < 0.05) (Fig. 3b). We hypothesized that there would be more false memories for negative images when compared to positive and neutral images. Planned comparisons revealed that there were significantly more false memories for negative images (Mdn = 6.2 %, IQR = 20.4) than for neutral images (Mdn = 0.0 %, IQR = 1.6) (T = 0.0, *p* < 0.05/2, one tailed). There was, however, no difference between positive (Mdn = 6.3 %, IQR = 14.1) and negative target images (T = 7.5, *p* > 0.05/2, one tailed).

### Secondary objectives - Effect of the cognitive profile of patients on the performance in the VR and recall tasks

Multiple linear regressions on the performance (%) during the VR task for neutral, positive and negative images, using MoCA and GDS scores as factors, showed significant models for positive (F(2,7) = 6.530, *p* < 0.05; MoCA: *p* > 0.05; GDS: *p* < 0.05; R^2^ = 0.651) and negative (F(2,7) = 7.282, *p* < 0.05; MoCA: *p* < 0.05; GDS: *p* > 0.05; *R*^*2*^ = 0.675) target images:

$$ \begin{array}{l}\mathit{\mathsf{Performance}}\_\mathit{\mathsf{V}}\mathit{\mathsf{R}}\_\mathit{\mathsf{Positive}}=\mathsf{82.00}+\mathsf{0.90}\cdot \mathit{\mathsf{M}\mathsf{oCA}}-\mathsf{1.16}\cdot \mathit{\mathsf{G}}\mathit{\mathsf{D}}{\mathit{\mathsf{S}}}^{*}\\ {}\mathit{\mathsf{Performance}}\_\mathit{\mathsf{V}}\mathit{\mathsf{R}}\_\mathit{\mathsf{Negative}}=\mathsf{15.80}+\mathsf{3.70}\cdot \mathit{\mathsf{M}}\mathit{\mathsf{o}}\mathit{\mathsf{C}}{\mathit{\mathsf{A}}}^{*}-\mathsf{1.58}\cdot \mathit{\mathsf{G}}\mathit{\mathsf{D}}\mathit{\mathsf{S}}\end{array} $$

These models reveal a significant negative effect of depressive symptomatology as assessed by the GDS on task performance for images with positive valence, as well as a significant positive effect of general cognitive functioning as assessed by the MoCA on task performance for images with negative valence.

In the case of the recall task, we observed that both MoCA and GDS scores could be used as predictors for the percentage of correctly recalled images (F(2,7) = 15.660, *p* < 0.01; MoCA: *p* < 0.05; GDS: *p* < 0.01; *R*^*2*^ = 0.817) and number of errors (F(2,7) = 23.969, *p* < 0.01; MoCA: *p* < 0.05; GDS: *p* < 0.001; *R*^*2*^ = 0.873) during the recall of neutral images:

$$ \begin{array}{l}\mathit{\mathsf{Performance}}\_\mathrm{R}\mathrm{e}\mathit{\mathsf{call}}\_\mathit{\mathsf{Neutral}}=\mathsf{59.50}+\mathsf{2.45}\cdot \mathit{\mathsf{M}}\mathit{\mathsf{o}}\mathit{\mathsf{C}}{\mathit{\mathsf{A}}}^{*}-\mathsf{3.97}\cdot \mathit{\mathsf{G}}\mathit{\mathsf{D}}{\mathit{\mathsf{S}}}^{*}\\ {}\mathit{\mathsf{Errors}}\_\mathrm{R}\mathrm{e}\mathit{\mathsf{call}}\_\mathit{\mathsf{Neutral}}=\mathsf{4.54}-\mathsf{0.25}\cdot \mathit{\mathsf{M}}\mathit{\mathsf{o}}\mathit{\mathsf{C}}{\mathit{\mathsf{A}}}^{*}+\mathsf{0.57}\cdot \mathit{\mathsf{G}}\mathit{\mathsf{D}}{\mathit{\mathsf{S}}}^{*}\end{array} $$

These models show specific effects of both depressive symptomatology and cognitive function for neutral images but not for images with positive or negative valence. Recall performance for neutral images is positively affected by cognitive function but negatively affected by depressive symptomatology. The opposite relationship is observed in the case of recall errors.

The inspection of the data for bivariate correlations between task (VR and recall) performance, MoCA subdomains and GDS showed additional contributions to the ones outlined by the models (Table 3). Focusing on the more relevant results, the performance in the VR task for negative images showed significant positive correlations with MoCA (*r* = 0.76, *p* < 0.05) and its Executive Functions (*r* = 0.75, *p* < 0.05), Naming (rho = 0.64, *p* < 0.05), Attention (*r* = 0.77, *p* < 0.05) and Orientation (*r* = 0.66, *p* < 0.05) subdomains. Moreover, the performance in the VR task for positive images showed a strong positive correlation with MoCA’s Memory subdomain (rho = 0.82, *p* < 0.01). Interestingly, there was a negative correlation with GDS meaning that the higher the depressive symptomatology the lower the performance, but only when the target images were positive (*r* = −0.65, *p* < 0.05). Concerning the performance in the recall task, in terms of the percentage of correctly recalled images, we observed a positive correlation with MoCA’s Memory subdomain (rho = 0.68, *p* < 0.05) and a negative correlation with GDS (*r* = −0.68, *p* < 0.05) when negative images were to be recalled. Finally, when analyzing the number of errors in the recall task (false negatives plus false positives), we observed a negative correlation with MoCA’s Memory subdomain (*r* = −0.64, *p* < 0.05) but for negative target images only.

**Table 3:** Analysis of correlations between scores in cognitive domains and performance in the VR and recall tasks

	VR task performance				Recall performance			Recall errors		
	Abstract	Neutral	Positive	Negative	Neutral	Positive	Negative	Neutral	Positive	Negative
MoCA - Total	0.64*	ns	ns	0.76*	0.63*	ns	ns	ns	ns	ns
MoCA - Exec Funct	ns	ns	ns	0.75*	ns	ns	ns	ns	−0.66*	ns
MoCA - Naming	0.65*	ns	ns	0.64*	ns	ns	ns	ns	ns	ns
MoCA - Attention	ns	ns	ns	0.77*	ns	ns	ns	ns	ns	ns
MoCA - Language	ns	ns	ns	ns	ns	ns	ns	ns	ns	ns
MoCA - Reasoning	ns	ns	ns	ns	ns	ns	ns	ns	ns	ns
MoCA - Memory	0.79**	ns	0.82**	ns	0.82**	ns	0.68*	ns	ns	−0.64*
MoCA - Orientation	ns	ns	ns	0.66*	ns	ns	ns	ns	ns	ns
GDS-30	ns	ns	−0.65*	ns	−0.78**	ns	−0.68*	0.86**	ns	ns

Correlation coefficients for two-tailed tests. *p < 0.05, **p < 0.01. ns: non-significant correlation

### Secondary objectives - Feasibility of the proposed VR rehabilitation paradigm

Our VR task assumes that there is a correspondence between VR and paper-and-pencil counterparts, and that the emotional content of images is perceived consistently by healthy individuals and stroke survivors.

#### Is performance on paper equivalent to VR?

In the original TP paper-and-pencil version, targets are always visible during performance. The paradigm was slightly simplified in the VR version to address the memory component and make it compatible with the presentation of emotional pictures (there is only one target symbol per trial, which is only visible for 2 s). Consequently, a comparison of the TP paper-and-pencil performance (Mdn = 60.0 %, IQR = 65.0) with the TP-VR performance (Mdn = 82.2 %, IQR = 30.4) revealed significantly better performance in VR when compared to traditional TP (T = 5.0, *p* < 0.05).

#### Do the patients’ ratings of the images differ from those of the original IAPS ratings for healthy individuals?

Overall, there was no significant difference between the original IAPS ratings (5.02 ± 2.37) and the participants reported ratings (5.01 ± 1.78). Nevertheless, when we analyzed separately the 3 emotional categories, we found significant differences between the ratings for the positive (IAPS = 7.85 ± 0.27, Patients = 6.75 ± 0.86; Mann–Whitney, U = 15.0, *p* < 0.001) and negative (IAPS = 2.15 ± 0.29, Patients = 3.15 ± 1.10; U = 36.5, *p* = 0.01), but not for neutral stimuli (IAPS = 5.07 ± 0.26, Patients = 5.14 ± 1.02; U = 73.0, *p* > 0.05). This indicates a consistent but more moderate rating of the emotional content in the images by our patients than those originally provided by IAPS.

## Discussion

There is growing evidence of the existence of cognitive-motor interference, and that motor and cognitive recovery need to be considered together [15]. Despite motor and cognitive stroke recovery patterns may differ, dual training has been shown to be effective for gait recovery [54], and there has also been shown a relationship of cognitive abilities such as reasoning and comprehension with functional motor performance, for the particular case of upper limb [55]. Here we presented a novel VR rehabilitation task that integrates both cognitive and motor domains. In this first study, we only addressed the cognitive component of the task by investigating the impact of emotional stimuli in task performance and eye-gaze behavior.

In this study, we assessed the impact of emotional stimuli in a group of ten stroke survivors. We used 3 types of stimuli: positive, negative and neutral. Additionally, we used abstract stimuli from the TP task to compare the VR task with its paper-and-pencil counterpart. A higher performance has been found in the VR version of the task, which can be explained by the use of only one target image (a less demanding cognitive task) and a natural user interface to facilitate the interaction between the patient and the VR environment (adapted motor challenge), eliminating some of the constraints of a paper-and-pencil task. This prevents situations in which the motor deficits, for instance in the dominant arm, may impede proper execution of the paper-and-pencil task.

The emotional ratings of the target images by our patients were consistent with those of the IAPS, but showed less extreme ratings for positive and negative valence. Even though ratings were more moderate, image valence had a measurable effect on task performance. We identified lower VR task performance for negative images than for neutral. This is consistent with the positivity effect described by the Socioemotional Selectivity Theory, which postulates that attention processes are less targeted to negative information in older adults [33]. Hence, given the average age of the general stroke population, this finding leads us to consider that positive and neutral content might be better for attention rehabilitation in this population.

In the case of the recall task, we observed that the lower performance for negative stimuli in VR was not replicated for overall performance during recall. These results are in accordance with the study by Steimetz and Kensinger [40] which shows that selective memory for emotional information is not strongly related to attention at encoding in healthy individuals. Interestingly, when we analyzed only the wrongly identified images -false positives- we found that the negative distractors led to significantly more mistakes. This finding is also consistent with the literature, with negative valence content causing more false memories [36], and a narrowing of attention at the cost of decreased memory for less salient details [38, 56]. This effect could also be observed in our study in several cases. For example, when having an image of a starving person as a target and then having two different images of a starving person in the recall task, the participant tended to select both, even if completely different. Overall, our findings lead us to conclude that using both positive and neutral stimuli will provide better results in the training of attention. Yet, if we want to increase task difficulty or memory challenge, we may consider negative stimuli since they are more difficult to remember and lead to more false memories. VR allows an easy customization depending on the objective and patient profile, which is more difficult to accomplish in traditional methods.

Another contribution of this study is the quantification of the relationship of patient profile -through the assessment of cognitive function and depressive symptomatology- and task performance depending on the emotional content of images. Our regression model analysis revealed that VR task performance for negative stimuli can be explained by the MoCA scores, being it a good metric for cognitive status. A positive relationship between cognitive function and performance with negative stimuli is consistent with increased difficulty of the task, as shown by VR task performance data. This finding is in agreement with our correlation analysis, which showed significant correlations of MoCA and multiple of its subdomains with overall VR performance. Of particular interest is the positive correlation of the memory subdomain of MoCA with VR performance for positive images. Further, a regression model showed that depressive symptomatology, as assessed by GDS-30, has a selective negative impact on VR task performance for positive stimuli. Consistent with the regression models, GDS-30 scores negatively correlated with performance for positive images. This finding is also in accordance with extensive literature that identified a general effect of negative mood in cognition in participants with mood disorders and depression [57, 58]. Interestingly, our models for the recall task capture effects of both cognitive function and depressive symptomatology, only for neutral stimuli and not for positive or negative. We also found a positive correlation of the memory subdomain of MoCA with recall performance, as well as a negative correlation with the number of errors for negative images. These data provide further evidence of distinct processing of emotional images in tasks involving attention, such as our VR task, in which the processing of negative stimuli is more strongly affected by overall cognitive function. Recall task performance correlates negatively with GDS-30 for negative images. Consequently, our data suggests that recall of positive stimuli is less modulated by cognitive function and depressive symptomatology, being it easier to recall than neutral or negative stimuli. This finding is also consistent with the shift towards emotional stimuli stated by the Socioemotional Selectivity Theory [33], and with the decline with age for negative stimuli [34]. Altogether, these findings take us to consider that, besides a rehabilitation purpose, this VR task making use of neutral, negative and positive stimuli may also be valuable in providing information about the patient’s mood and cognitive status.

Fixations and saccades in our VR task are most likely related to a visual search component, whereas smooth pursuit segments may be more related to eye-hand coordination [59]. Compared to previous eye tracking research on action observation and execution in VR with stroke survivors [23, 27], our data shows shorter fixations, smooth pursuits and longer saccades. These changes are probably associated to an increase of visual search patterns due to the existence of multiple distractors in our task. The eye tracking density maps show more disperse gaze patterns for neutral than for emotionally charged stimuli, supporting the premise of an attentional shift towards stimuli with emotional content. More concretely, our data shows differences in eye gaze metrics only for incorrect choices and not for correct ones. We observed lower count of saccades for positive stimuli, and shorter trajectories for negative stimuli. Hence, eye gaze metrics are suggestive of more determined and direct responses and with shorter search patterns for stimuli with positive or negative valence than for neutral. This information complements the available VR task performance data, which indicated more errors for negative stimuli. These data support the idea that errors in the VR task are the result of encoding the emotional content of the images, while neglecting peripheral non-emotional information.

## Conclusions

In this study, distinct effects of overall cognitive function and mood were observed for images with neutral, positive and negative valence, for both attention and memory recall. These results contribute towards understanding how emotional content of images can be used on a VR paradigm for tailoring stimuli in cognitive-motor rehabilitation to each patient profile. However, a deeper understanding of the role of the motor component of the task needs to be developed. For this reason, we are currently investigating the effect of this VR task using positive valence in a 1-month randomized controlled longitudinal intervention, with pre and post motor assessment.

## Abbreviations

ADL, Activities of Daily Living; GDS, Geriatric Depression Scale; IAPS, International Affective Picture System; LB, Line Bisection; MoCA, Montreal Cognitive Assessment; SAM, Self-Assessment Manikin; SIS, Stroke Impact Scale; TP, Toulouse Piéron; VR, Virtual Reality
